# Comparison of Clinical Outcomes between Idiopathic Frozen Shoulder and Diabetic Frozen Shoulder After a Single Ultrasound-Guided Intra-Articular Corticosteroid Injection

**DOI:** 10.3390/diagnostics10060370

**Published:** 2020-06-04

**Authors:** Chul-Hyun Cho, Hyo-Joon Jin, Du Hwan Kim

**Affiliations:** 1 Department of Orthopedic Surgery, Dongsan Medical Center, School of Medicine, Keimyung University, Daegu 42601, Korea; oscho5362@dsmc.or.kr; 2 Department of Rehabilitation Medicine, Dongsan Medical Center, School of Medicine, Keimyung University, Daegu 42601, Korea; gywns9212@naver.com; 3 Department of Physical Medicine and Rehabilitation, College of Medicine, Chung-Ang University, Seoul 06973, Korea

**Keywords:** bursitis, intra-articular injections, diabetes mellitus, adrenal cortex hormones

## Abstract

There is no consensus on the use of intra-articular corticosteroid injections in diabetic frozen shoulder (FS). Thus, we aimed to compare clinical outcomes after intra-articular corticosteroid injections in patients with diabetic FS and idiopathic FS. Data collected from 142 FS patients who received glenohumeral joint intra-articular corticosteroid injections were retrospectively reviewed. Thirty-two patients were diagnosed with diabetic FS and 110 patients with idiopathic FS. Data including visual analog scale (VAS) for pain, American Shoulder and Elbow Surgeons (ASES) score, subjective shoulder value (SSV), and passive range of motion (ROM) were compared before the injection and at 3, 6, and 12 weeks after the injection. There were significant improvements in all outcomes (*p* < 0.001 for all parameters) through 12 weeks in both groups. There were no significant differences in all outcomes, except for ASES scores, between both groups at 3 weeks. However, there were significant differences in VAS score, SSVs, ASES scores, and passive ROMs, except for angle of abduction, between the two groups at 6 weeks and 12 weeks after injection. A single intra-articular steroid injection can be used as a conservative treatment for diabetic FS, but less effective than for idiopathic FS.

## 1. Introduction

Frozen shoulder (FS) is a common shoulder disorder characterized by progressive painful stiffness of the glenohumeral joint that is caused by an inflammatory contracture of the capsule [1,2]. FS usually develops without any trauma or specific shoulder disease [3]. If the cause of the painful stiff shoulder is unknown, then “idiopathic FS” is considered.

Although the exact pathogenesis of FS is unknown, the risk factors include female sex, diabetes, thyroid disease, and hypercholesterolemia [4,5,6,7,8]. Among several risk factors, diabetes is suggested to be one of the strongest factors for the development of FS. If patients with a painful stiff shoulder are diagnosed with diabetes, the term “diabetic FS” is commonly used [8,9,10]. The prevalence of FS in patients with diabetes (28–40%) is higher compared to the general population (3–5%) [1,4,6]. Further, the response to treatment in patients with diabetic FS is poorer than that of non-diabetic FS [1,4,6]. In idiopathic FS, intra-articular corticosteroid injection is commonly used as a conservative treatment, especially at the inflammatory stage is dominant. A few studies suggested that intra-articular corticosteroid injection can be a useful treatment option even for diabetic FS [11,12]. The number of patients with diabetes has rapidly increased due to the extended lifespan and sedentary lifestyle. Although clinicians will inevitably encounter more patients with diabetic FS in the future, there is no consensus as to the use of intra-articular corticosteroid injection in diabetic FS. The role of the intra-articular steroid injection in diabetic FS needs to be clearly explored. To our knowledge, few studies have been conducted to compare the serial changes in pain, functional scores, and range of motion (ROM) after intra-articular corticosteroid injections between patients with and without diabetes. The purpose of this study was to compare serial outcomes after intra-articular corticosteroid injections in patients with diabetic FS and idiopathic FS. We hypothesized that patients with diabetic FS might respond to intra-articular corticosteroid injections, but these injections would be less effective for pain, functional scores, and ROMs during the short-term period compared with patients with idiopathic FS.

## 2. Materials and Methods

### 2.1. Patients

**Figure 1:**
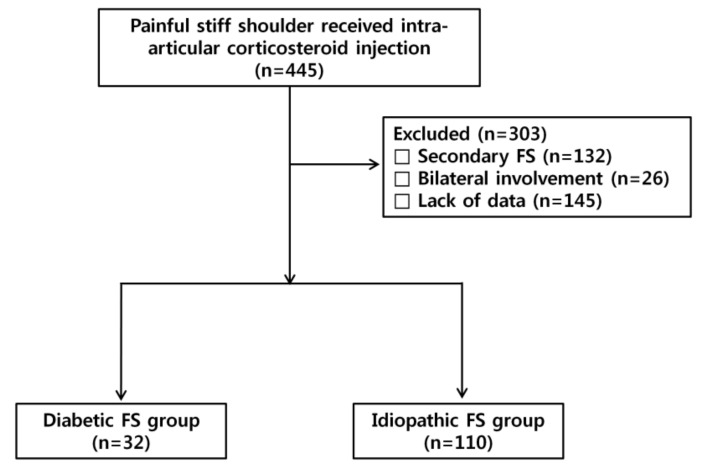
Flowchart illustrating patient selection and the number of patients.

We retrospectively reviewed prospectively collected data of 445 consecutive patients with FS who received intra-articular corticosteroid injections in the glenohumeral joint under a single physiatrist from March 1, 2014 until July 31, 2017. In total, 132 patients received intra-articular corticosteroid injection because of secondary frozen shoulders, such as rotator cuff-related stiffness, osteoarthritis, or rheumatic diseases, and were excluded from the study. Twenty-six patients with bilateral involvement were excluded, and 145 patients were subsequently excluded due to the lack of follow-up data. The remaining 142 patients with the diagnosis of FS were dichotomized into idiopathic FS group and diabetic FS group (Figure 1). All 142 patients dichotomized as idiopathic or diabetic FS group underwent plain radiography and ultrasonography (US) or magnetic resonance imaging to detect secondary causes for painful stiffness. The inclusion criteria were (1) unilateral shoulder pain with limitations of passive motion in two or more planes movement (abduction and forward flexion <130 degrees, external rotation <45 degrees, or internal rotation <L1) on the baseline check-up and (2) normal plain radiography. The exclusion criteria were (1) secondary FS, such as the concomitant rotator cuff tear, calcific tendinitis, and rheumatic diseases, (2) infection, (3) osteoarthritis, (4) history of high-energy trauma, (5) previous shoulder surgery, (6) previous corticosteroid injection on the affected side within 3 months before the visit of our clinic, (7) incomplete data before the injection and at 3, 6, and 12 weeks after the injection, and (8) poor cognitive function. This study was approved by Dongsan Medical Center Institutional Review Board (IRB No. 2017-09-022-0010).

### 2.2. Treatment Protocol

The method of injection was a posterior approach using US-guidance. The injection was performed with the patient in the semi-lateral decubitus position on the unaffected side with anterior tilting of the affected side at 45 degrees. The needle was advanced from the lateral to medial side with the visualization of the needle shaft under US-guidance using a linear 5 to 12 MHz probe (HD15 ultrasound system; Philips) and reached the glenohumeral joint space between the posterior humeral head and posterior glenoid labrum. The injection mixture for both groups consisted of 40 mg of triamcinolone acetonide, 4 mL of 1% lidocaine, and 4 mL of normal saline. All injection procedures were performed by a single physiatrist blind to clinical findings.

All patients were instructed to follow a home-based stretching exercise program to increase ROMs and were encouraged to perform a home-based stretching exercise three times a day (15 min each round). A home-based exercise program included pendulum exercises, wall-climbing stretch exercises (place palm against wall and climb wall as high as possible with fingers), and gentle ROM exercises with a bar. During the home exercise program, patients were asked to stretch their shoulder within the ROMs without provoking post-mobilization soreness with self-feedback. Patients were not allowed to receive acupuncture or additional injections from other hospitals.

### 2.3. Outcome Assessment

Data including the visual analog scale (VAS) for pain, American Shoulder and Elbow Surgeons (ASES) score, subjective shoulder value (SSV), and passive ROMs [13,14] were collected prospectively before the injection and at 3, 6, and 12 weeks after the injection. The ROMs, including forward flexion, abduction, and external rotation were assessed by a goniometer in the sitting position. Angles of forward flexion and abduction were evaluated including the scapulohumeral motion. To measure the internal rotation ROM, a scratch test was performed by recording the vertebral level reached with the tip of the thumb in the sitting position. The vertebral level was then converted into a serial number as follows: T1–T12 into 1–12, respectively; L1–L5 into 13–17; sacrum into 18; coccyx into 19; and buttocks into 20. The measurement of clinical outcomes was conducted by another physician who was blind to the presence of diabetes.

### 2.4. Statistical Analysis

Demographic factors at baseline were compared between the two groups using the Mann-Whitney *U* test and the chi-square test. For both groups, repeated measures ANOVA was used to determine if each outcome had a time effect after injection. The Mann-Whitney *U* test was used to compare the differences between the outcomes of two groups at each point. A *p* value of <0.05 was considered significant. Statistical analysis was performed using the SPSS program (SPSS 18.0, Chicago, IL, USA).

## 3. Results

### 3.1. Patients’ Characteristics

This study comprised 32 patients with diabetic FS and 110 patients with idiopathic FS. All patients enrolled in diabetic FS group were type II diabetes. At baseline, there were no significant differences in age, the involvement of dominance side, duration of symptoms and clinical scores between the two groups (Table 1). However, the proportion of females was significantly higher in the idiopathic FS group than in the diabetic FS group (*p* = 0.039).

### 3.2. Changes in Outcome Measurements for Patients with Diabetic or Idiopathic FS

**Figure 2:**
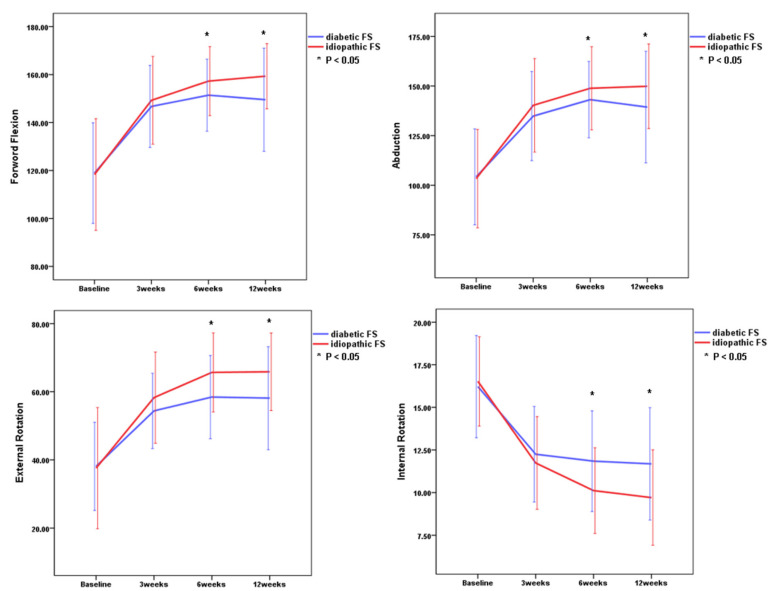
Comparison of range of motion between the two groups. Internal rotation was recorded on the basis of the vertebral level reached with the tip of the thumb. Asterisk indicates significant differences between the idiopathic frozen shoulder group and the diabetic frozen shoulder group at each time point. Error bar means one standard deviation.

**Figure 3:**
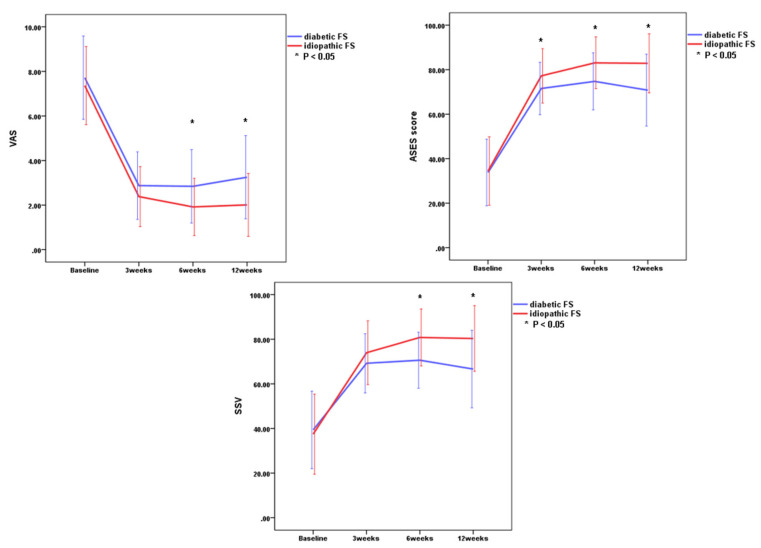
Comparison of functional scores (VAS, ASES score, SSV) between the two groups. Asterisk indicates significant differences between the idiopathic frozen shoulder group and the diabetic frozen shoulder group at each time point. VAS, visual analog scale; ASES, American Shoulder and Elbow Surgeon score; SSV, subjective shoulder value. Error bar means one standard deviation.

There were significant improvements in all outcome measurements (*p* < 0.001 for all parameters) including VAS scores, ASES scores, SSVs, and passive ROMs through 12 weeks in both idiopathic FS group and diabetic FS group (Table 2 and Figure 2 and Figure 3). These results indicated that all outcome measurements at 3, 6, and 12 weeks after the injection were significantly improved as compared to baseline in both groups. There were greatest improvements of all parameters within the first 3 weeks in both groups. 

### 3.3. Comparison of Clinical Outcomes Between Idiopathic and Diabetic FS at Each Point

There were no significant differences in VAS score, SSVs, and passive ROMs between the idiopathic FS group and the diabetic FS group at 3 weeks, but there was a significant improvement of ASES in the idiopathic FS group when compared with the diabetic FS group at 3 weeks (*p* = 0.027) (Table 3 and Figure 1 and Figure 2). There were significant differences in VAS score, SSVs, ASES scores, and passive ROMs, except for the angle of abduction, between the idiopathic FS group and the diabetic FS group at 6 weeks and 12 weeks after injection (Table 3 and Figure 2 and Figure 3). These results indicated that the idiopathic FS group had more improvements in all outcome measurements, except for abduction ROM, than the diabetic FS group at 6 and 12 weeks after the injection.

No patient reported serious side effects, such as infections, necrosis, vasovagal syncope, systemic toxicity of the local anesthetic, or anaphylactic response. Seven patients complained of temporary facial flushing, and two patients complained of skin itching sensation.

## 4. Discussion

This present study revealed that intra-articular corticosteroid injection led to significant improvements in pain severity, functional scores, and ROMs through 12 weeks in both diabetic and non-diabetic FS patients. There were no significant differences in all parameters between the diabetic group and the non-diabetic group at 3 weeks after injection except for ASES score, whereas there were significant differences in all parameters except for abduction ROM between the diabetic group and the non-diabetic group at 6 weeks and 12 weeks after injection. These results suggest that diabetes can affect the outcomes after intra-articular steroid injection in FS.

It has been widely-accepted by some experts that the treatment of diabetic patients with FS compared to that of idiopathic FS is more difficult and they show more resistance to treatment [15,16,17,18,19,20,21,22,23]. However, there have been few well-designed studies of natural history of diabetic FS and comparison of clinical outcomes between diabetic FS and idiopathic FS after conservative treatments [14,24]. Also, the concept of resistance to treatment in diabetic FS has been mainly based on the results of studies about arthroscopic capsular release or manipulation under anesthesia [10,16,18,20,25,26,27,28]. Rill et al. reported that patients with diabetes had a lower final Simple Shoulder Test score than patients without diabetes after non-operative treatments which were heterogeneous to each patient, but diabetes did not predict the need for surgery [29]. Dehghan et al. reported that intra-articular steroid injection is effective in the treatment of diabetic FS, but there is no significant difference in efficacy between intra-articular steroid injection and non-steroidal anti-inflammatory drugs [11]. Roh et al. also demonstrated that intra-articular steroid injection led to more improvement in pain and functional score in the early post-injection period compared to the home stretching treatment, but there were no significant differences at 24 weeks after injection between intra-articular corticosteroid injection and home stretching group [12]. Although other confounding factors, such as uncontrolled treatment protocol, could explain the discrepancy between the results for diabetic patients and patients with idiopathic FS, there has been no report on the direct comparison of the effects of intra-articular corticosteroid injections in diabetic patients and non-diabetic patients. This present study revealed that a single intra-articular corticosteroid injection led to significant improvements in all outcome measurements including VAS scores, ASES scores, SSVs, and passive ROMs through 12 weeks in both the idiopathic and diabetic FS group, but the improvement of all parameters in the idiopathic FS group was significantly higher than that in the diabetic FS group at 6 and 12 weeks. These results indicate that a single intra-articular steroid injection can be used as a conservative treatment of diabetic FS, but its effect in diabetic FS is less than in idiopathic FS. This finding is suggestive of a limited role of intra-articular steroid injection in diabetic FS.

It is unclear why there were no significant differences in most parameters between the diabetic group and the non-diabetic group at 3 weeks after injection, whereas there were significant differences in all parameters except for abduction ROM between the diabetic group and the non-diabetic group at 6 weeks and 12 weeks after injection. We postulated that the characteristic of diabetic FS capsule would be, histologically, somewhat different from that of non-diabetic FS capsule [8,21,30,31]. At 3 weeks, intra-articular steroid injection mainly acts on synovial inflammation component leading to a reduction of inflammation or vascular hyperplasia in both idiopathic and diabetic FS group. Kabbabe et al. described that comparison between diabetic and non-diabetic patients with FS revealed a decrease in the level of expression of inflammatory cytokine, monocyte colony-stimulating factor in diabetic FS patients [32]. Considering Kabbabe’s study, the attenuated responses of intra-articular steroid injection at 6 and 12 weeks after injection in diabetic FS might be related to decreased expression of inflammatory cytokines [32]. Other differences between idiopathic and diabetic FS included greater endothelial growth factor levels and abnormal collagen cross-linking and following fibrosis in the latter [21,33,34,35]. All these differences account for the attenuated response to intra-articular steroid injection treatment in diabetic FS. The results of our study suggest that diabetic FS differs from idiopathic FS in some respects, but the mechanism underlying the different responses of both groups to intra-articular steroid injection could not be fully explained by previous studies’ results. Basic science research on what causes these findings is needed and can provide a treatment alternative for diabetic FS.

This study has several limitations. First, the number of enrolled patients, especially diabetic patients, was small. Second, we did not survey the HbA1C level at baseline and monitor the change of blood control method, fructosamine, and HbA1C level after injection in diabetic patients. Third, we did not evaluate long-term effects after injection. Considering the pharmacokinetics, we presumed that the intra-articular corticosteroid injection mainly has a short-term effect. Fourth, we did not assess the compliance with home exercise even though exercise could affect the outcomes. Lastly, the proportion of females was higher in the idiopathic FS group than in the diabetic FS group. Idiopathic FS group in this present study reflects the general concept that idiopathic FS has female dominance. This sex disproportion factor may affect the outcomes.

## 5. Conclusions

Intra-articular corticosteroid injection led to significant improvements in pain severity, functional scores, and ROMs throughout 12 weeks of monitoring in both diabetic and idiopathic FS patients. However, the effects in the diabetic FS group were attenuated at 6 and 12 weeks when compared with the idiopathic FS group. A single intra-articular steroid injection can be used as a conservative treatment of diabetic FS, but its effect in diabetic FS is less than that in idiopathic FS.

## Figures and Tables

**Table 1:** Baseline demographics of patients with diabetic or idiopathic FS.

Variable	Diabetic FS Group	Idiopathic FS Group	*p*-Value
Number of patients	32	110	
Age	56.8 ± 8.2	56.6 ± 8.6	0.497
Male:female (no.)	18:14	38:72	0.039
Right:left (no.)	13:19	52:58	0.550
Duration of symptoms (months)	7.2 ± 6.0	6.8 ± 6.8	0.364
Initial clinical score			
VAS	7.7 ± 1.8	7.3 ± 1.7	0.276
ASES	33.8 ± 14.9	34.4 ± 15.3	0.698
SSV	39.4 ± 17.4	37.4 ± 17.9	0.546
Initial ROM			
Forward flexion	118.9 ^o^ ± 20.9°	118.3 ^o^ ± 23.2°	0.933
Abduction	104.2 ^o^ ± 24.1°	103.3 ^o^ ± 24.7°	0.747
External rotation	37.7 ^o^ ± 12.0°	37.8 ^o^ ± 16.8°	0.706
Internal rotation ^†^	16.2 ± 3.0	16.5 ± 2.6	0.419

FS, frozen shoulder; VAS, visual analog scale; ASES, American Shoulder Elbow Surgeons; SSV, Subjective Shoulder Value; ROM, range of motion. Values are given as the mean and SD, except for the number of patients, sex ratio, and ratio of involved side. ^†^ T1–T12 into 1–12, respectively; L1–L5 into 13–17; sacrum into 18; coccyx into 19; and buttocks into 20.

**Table 2:** Serial changes in outcome measurements for patients with diabetic or idiopathic FS.

	Baseline		3 Weeks		6 Weeks		12 Weeks		Time Effect (*p*-Values)	
	Diabetic FS Group	Idiopathic FS Group	Diabetic FS Group	Idiopathic FS Group	Diabetic FS Group	Idiopathic FS Group	Diabetic FS Group	Idiopathic FS Group	Diabetic FS Group	Idiopathic FS Group
VAS	7.7 ± 1.8	7.3 ± 1.7	2.8 ± 1.5	2.4 ± 1.3	2.8 ± 1.6	1.9 ± 1.3	3.3 ± 1.9	2.0 ± 1.4	<0.001	<0.001
ASES	33.8 ± 14.9	34.4 ± 14.3	71.6 ± 11.8	77.2 ± 12.2	74.7 ± 12.8	83.1 ± 11.6	70.8 ± 16.2	82.8 ± 13.3	<0.001	<0.001
SSV	39.4 ± 17.4	37.4 ± 17.9	69.2 ± 13.3	74.0 ± 12.2	70.6 ± 12.6	80.8 ± 12.8	66.6 ± 17.4	80.4 ± 14.8	<0.001	<0.001
FF	118.9° ± 20.9°	112.3° ± 23.1°	146.7° ± 17.1°	149.3° ± 18.3°	151.4° ± 15.0°	157.3° ± 14.4°	149.5° ± 21.5°	159.3° ± 13.6°	<0.001	<0.001
ABD	104.7° ± 24.1°	103.3° ± 24.7°	134.8° ± 22.5°	140.3° ± 23.6°	143.1° ± 19.3°	148.9° ± 21.0°	139.4° ± 28.2°	149.9° ± 21.4°	<0.001	<0.001
ER	37.7° ± 12.0°	37.8° ± 16.8°	54.4° ± 11.1°	58.3° ± 13.4°	58.4° ± 12.2°	65.7° ± 11.6°	58.1° ± 15.1°	65.9° ± 11.4°	<0.001	<0.001
IR	16.2 ± 3.0	16.5 ± 2.6	12.3 ± 2.8	11.7 ± 2.7	11.8 ± 3.0	10.1 ± 2.5	11.7 ± 3.3	9.7 ± 2.8	<0.001	<0.001

FS, frozen shoulder; US, ultrasonography; VAS, visual analog scale; ASES, American Shoulder Elbow Surgeons; SSV, Subjective Shoulder Value; FF, forward flexion; ABD, abduction; ER, external rotation; IR, internal rotation (T1–T12 into 1–12, respectively; L1–L5 into 13–17; sacrum into 18; coccyx into 19; and buttocks into 20).

**Table 3:** Statistical analysis of outcome measurements at each point for patients with diabetic or idiopathic FS.

	VAS	ASES	SSV	FF	ABD	ER	IR
Baseline	0.276	0.698	0.546	0.933	0.747	0.706	0.419
3 weeks	0.110	0.027 *	0.065	0.322	0.172	0.128	0.392
6 weeks	0.003 *	0.001 *	<0.001 *	0.034 *	0.096	0.008 *	0.002 *
12 weeks	<0.001 *	0.001 *	<0.001 *	0.021 *	0.066	0.009 *	0.002 *

FS, frozen shoulder; VAS, visual analog scale; ASES, American Shoulder Elbow Surgeons; SSV, Subjective Shoulder Value; FF, forward flexion; ABD, abduction; ER, external rotation; IR, internal rotation. * statistically significant.
